# Controlling Factors of Degassing in Crosslinked Polyethylene Insulated Cables

**DOI:** 10.3390/polym11091439

**Published:** 2019-09-02

**Authors:** Dong Joon Youn, Jingfa Li, Sara Livazovic, Yabin Sun, Shuyu Sun

**Affiliations:** 1 King Abdullah University of Science and Technology, Thuwal 26955, Saudi Arabia; jingfa.li@kaust.edu.sa; 2 Dow Middle East Innovation Center, Dow Material Science, Thuwal 26955, Saudi Arabia; SLivazovic@dow.com; 3 Dow Chemical (China) Investment Co., Ltd., Shanghai 201203, China; SYSun@dow.com

**Keywords:** crosslinked polyethylene (XLPE) insulation, methane degassing, Fick’s law of diffusion, temperature-dependent diffusion, cable conductor, nonuniform concentration

## Abstract

Here, we analyze the degassing process of a byproduct (methane) formed during the peroxide-induced crosslinking of polyethylene. A diffusion model based on Fick’s law is used to obtain the controlling factors of degassing in a crosslinked polyethylene (XLPE) insulated power cable (132 kV with 18 mm of insulation). We quantitatively analyze different scenarios of the diffusion of methane through the XLPE insulation and two semiconductor layers under various in situ degassing conditions. The analyzed degassing conditions include heat transfer and its effect on the diffusion properties, the different transport and boundary conditions due to the free spaces within the cable conductor, and the nonuniform distribution of methane concentrations within the insulation layers. Our simulation results clearly demonstrate that the free spaces between the copper strands in the cable conductor significantly affect the degassing efficiency. However, the temperature-diffusion coupling has a relatively minor effect on the overall degassing efficiency due to the rapid temperature increase of the polymer layers during the initial stages of degassing. Moreover, we find that the nonuniform distribution of methane in the initial stages also plays an important role in degassing in the cable, but this effect varies significantly during the degassing process.

## 1. Introduction

Power cables are an essential part of modern urban power transmission and distribution [1,2]. The representative structure of such cables is mostly typically comprised of a conductor core, insulation, and a shield/jacket. The insulation plays a crucial role in determining the quality and properties of the cable, especially for high-voltage (HV) and extra-high-voltage (EHV) cables. As an alternative technique to the traditional impregnated paper-oil insulation, polyethylene (PE) is globally preferred as cable insulation due to its easy processing properties, good electrical and thermo-mechanical behaviors, and relatively low cost [3]. Chemical crosslinking is commonly performed on PE during the cable extraction process. This has led to the development of crosslinked polyethylene (XLPE), which provides improved mechanical properties such as structural integrity, abrasion, and chemical resistance, especially at high temperatures.

In the cable industry, peroxide-induced crosslinking is one of the most popular and easiest approaches. Dicumyl peroxide (DCP) is usually used as a highly-efficient crosslinking agent due to its desirable decomposition rate at normal processing temperatures. During the crosslinking process, however, several byproducts can be generated and can remain within the XLPE. Typical unwanted byproducts include methane, acetophenone (AP), and cumyl alcohol (CA) [4,5,6]. The presence of these byproducts has a detrimental impact on a cable’s quality and long-term properties; they not only affect the performance of cables and their associated properties (e.g., by increasing the internal pressure and reducing the integrity of the insulation/splice interface), but they also cause significant environmental and safety issues due to the flammability of methane [7,8,9,10]. Thus, an efficient reduction in peroxide-related byproducts is a crucial factor in the manufacturing process to prevent excessive accumulation of unwanted byproducts, especially methane and its associated combustion risk.

To reduce byproduct concentration levels properly within the XLPE insulation layer, thermal treatment is recommended during the cable production process. This process, known as byproduct degassing, takes place after the insulation layer has cooled down and has solidified in the continuous vulcanization line. The produced cable is coiled on reels, transported to the degassing chambers, and heated to a specific temperature for approximately one week to remove methane and other byproducts from the XLPE insulation [9,11,12]. Degassing is an expensive and time-consuming process, which depends on the time that the byproducts migrate through the XLPE insulation and are released to the air. To improve and optimize the degassing process, the mechanism of the diffusion of the byproducts from the XLPE insulation should be comprehensively studied and understood. In the past decades, there have been several studies focusing on the degassing process via experimental and numerical approaches. Several analytical methods were employed for such analytical studies, such as gas chromatography (GC), Raman spectroscopy, thermo-gravimetric analysis (TGA), differential scanning calorimetry (DSC), high-pressure liquid chromatography (HPLC), Fourier transform infrared spectroscopy (FTIR), etc. In the CIGRE TB501 standard [12], the current state-of-the-art on these methods was described in detail, including the precision and reliability of these methods. We briefly review some representative works on byproduct measurement experiments. Smedberg et al. [13] performed several analytical experiments such as TGA, GC, and FTIR line scan to measure the amount of degassing and compared the advantages and drawbacks of these measurement techniques. Kolley [14] tested multiple experimental methods and combined HPLC with an ultraviolet detector to improve the measurement of byproducts. To account for changes in diffusion with changing temperature, Kemper et al. [15] proposed an enhanced measurement technique by applying large-spot Raman spectroscopy and monitored the quantities of CA and AP. Sahyoun et al. [16] presented a study on the diffusion mechanism under different experimental conditions, i.e., the diffusion coefficient of each byproduct as a function of desorption conditions and byproduct characteristics. Ji et al. [17] developed a multiple headspace gas bag method that combined the techniques of gas sampling and multiple headspace extraction. Compared with conventional methods, the advantage of the multiple headspace gas bag method is that it minimizes methane loss during sample preparation.

Although experimental measurements are of great importance for understanding the complex degassing process, it is not practical to implement all kinds of experiments for different cable designs, production conditions, degassing parameters, etc. Alternatively, the numerical simulation technique is a powerful tool to supplement experiments and achieve a fundamental understanding of the degassing process. The diffusion behaviors of the byproducts are influenced by various factors and parameters, such as initial concentrations, degassing temperature, cable design, etc. Andrews et al. [9] described the changing of byproduct concentrations mathematically using Fick’s law. The finite element-based approach was applied to solve the diffusion equation, and a number of calculations were used to examine the effect of different cable geometries, static temperature gradients, and multilayer structures on the degassing time. Christen [18] developed a diffusion model by deriving a Smoluchowski equation and defined the effect of the spurious drift on diffusion in the solid polymers, which showed an Arrhenius temperature-dependent mobility. Vissouvanadin et al. [19] proposed a diffusion model considering the effect of a heterogeneous polarization charge on the byproduct diffusion process. Smedberg et al. [20] presented a study on the degassing process of HV XLPE-insulated cables and numerically investigated the influence of CA and AP on the electrical properties of the cable. Sun et al. [11] and Sun and Person [21] applied a finite slab diffusion model to account for the degassing process of a small XLPE sample used in laboratory experiments. Sahyoun et al. [16] derived a mathematical expression for a plane sheet of constant thickness with the surfaces maintained at a constant byproduct concentration based on Fick’s diffusion laws in sorption or desorption mode.

In recent years, there has been an ever-increasing demand for HV and EHV power cables, with equal demand that the cable manufacturing process meet technical requirements, as well as safety and environmental standards. However, with a thicker XLPE insulation layer, degassing becomes an increasingly critical bottleneck for cable production. As the studies mentioned above highlighted, the degassing of byproducts from XLPE-insulated cables has drawn considerable attention from many researchers and cable manufacturers. However, due to the complicated effects of various changing parameters on the diffusion behaviors of byproducts, much remains to be clarified on the degassing process of byproducts, especially for methane, and more analysis is needed. In previous works, cable geometry was simplified into a slab model, the nonuniform initial concentration of byproducts in fresh cables was ignored, and there was no consideration for the changes in the diffusion coefficient due to temperature variations in the degassing chamber.

To obtain a more complete understanding of the degassing process and to explore more efficient degassing conditions, we performed a series of comprehensive parametric studies on the degassing process, as well as on the influence of the geometrical characteristics of the cables and the degassing chambers. Our simulation results described in this paper show how varying conditions in the cable insulation and degassing chamber affect byproduct transport and the overall degassing efficiency. In Section 2, we present the details of the computational method, including numerical assumptions, governing equations, cable geometry, and modeling conditions. In Section 3, we discuss the degassing simulation results with respect to various degassing conditions, such as the temperature-dependent diffusion coefficient, the effect of internal holes in the cable conductor, the initial nonuniform methane concentration, and their quantitative effect on degassing. Our conclusions and suggestions for future research regarding byproduct degassing are presented in Section 4.

## 2. Computational Model

We calculated the methane concentration change over time based on Fick’s law of diffusion by using a finite element-based approximation. Several assumptions were firstly made to account for the methane transport mechanism and the in situ degassing chamber conditions efficiently and reliably, as outlined below.

- The XLPE and semiconductor are homogeneous and isotropic in terms of the methane diffusion and heat transfer.
- Methane transport is solely governed by the conventional Fick’s laws with the concentration-independent diffusion coefficient and the concentration gradient of the methane.
- The XLPE and semiconductors stick tightly together, and thus, there is no free space between the layers.
- The radial flow of the methane is much larger than the longitudinal flow during cable degassing due to the much greater cable length scale (over 500 m) compared to the cross-section (the diameter is less than 10 cm).
- The methane concentration in the degassing chamber is always close to zero due to the large volume of the degassing chamber and the sufficient amount of fresh air intake.

Due to the significantly different scales of the cross-section and length of the power cables, almost all the methane must be transferred along the radial direction, and the longitudinal methane flow does not greatly contribute to the degassing process or its efficiency. Therefore, we used a two-dimensional cable geometry in our degassing simulations, which is representative of methane diffusion occurring in the middle of the cable.

### 2.1. Governing Equations

Methane transport in an XLPE insulated cable is commonly assumed to be a diffusional flow due to the relatively low concentration levels and the transport efficiency of the byproducts within the polymer layers. Reduction of the byproduct concentration from an XLPE cable, therefore, can generally be represented by Fick’s law, as in Equations (1) and (2) [9,13,16,17], which is based on the assumptions that the diffusion flux is proportional to the concentration gradient and the rate of concentration change is proportional to the second derivative of concentration with space.

(1) J=−D∇c

where *J* is the diffusion flux, *D* is the diffusion coefficient, and *c* is the concentration of fluid under consideration, i.e., methane in this work. Combined with the continuity equation, Equation (1) is extended to Fick’s second law, as shown in Equation (2), which is a parabolic linear partial differential equation to estimate how the concentration changes with time and the diffusion coefficient, *D*.

(2) $$\frac{\partial c}{\partial t}=D\nabla^{2}c$$

where *t* is the time. The diffusion coefficient, *D*, heavily depends on temperature *T*, and the temperature-dependent variation is commonly defined by the Arrhenius equation as in Equation (3) [16,21].

(3) $$D(T)=D_{0}\exp\left(\frac{-E_{a}}{RT}\right)$$

where $D_{0}$ is a dimensionless pre-exponential factor defining the quantitative relation of the measured diffusion at various temperatures, $E_{a}$ is the activationenergy, and *R* is the universal gas constant, 8.314 J/mol K.

For the estimation of temperature changes in the cable over time, the governing equation of heat transfer is derived as in Equation (4) from the law of thermodynamics, by assuming that the heat transfer of the cable is independent of the mechanical behavior.

(4) $$\rho C_{p}\frac{\partial T}{\partial t}=\nabla \cdot q+\nabla \left(k\nabla \cdot T\right)$$

where ρ is the density, $C_{p}$ is the specific heat capacity at constant pressure, q is the heat flux, and *k* is the heat conductivity. We also assumed that the cable materials were isotropic with constant thermophysical properties, but without any volumetric sources. Hence, Equation (4) is rewritten as in Equation (5).

(5) $$\frac{\rho C_{p}}{k}\frac{\partial T}{\partial t}=\nabla^{2}T$$

For thermal-diffusion coupling analysis, the heat transfer analysis was firstly carried out using Equation (5). The calculated temperature data were then applied to Equation (3) to update the diffusion coefficient, and later, the recalculated diffusion coefficient was applied to Equation (2) to approximate the concentration change at a monitoring time. This iteration process was repeated at each predefined time step up to the total degassing time, which is usually 7–9 days [9,16].

### 2.2. Cable Geometry and Modeling Conditions

**Figure 1:**
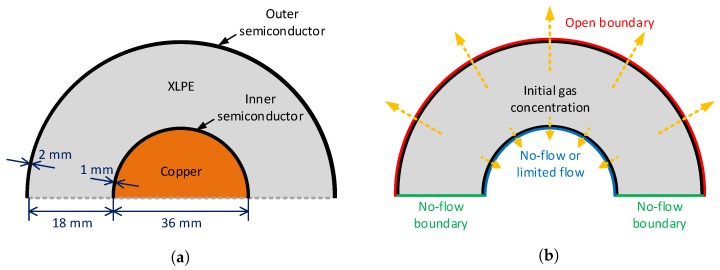
A two-dimensional schematic representation of a 132-kV HV cable with an 18-mm XLPE insulation: (**a**) geometry and (**b**) initial and boundary conditions for the methane diffusion.

To perform representative degassing analyses, we first selected a single core HV cable with typical XLPE insulation configuration, a 132-kV power cable with an 18-mm XLPE insulation layer. The cable included a conductor of 36 mm in diameter located at the cable center, and the conductor was sequentially surrounded by additional polymer layers, namely inner semiconductor (ISC), XLPE, and outer semiconductor (OSC). The 132-kV HV cable geometry is demonstrated in Figure 1a.

The initial concentration of methane was set to 28.68 mol/m${}^{3}$ (equivalent to 500 ppm), and this quantity was uniformly applied within the XLPE layer for all of the initial parametric studies, except for cases where a set of specific nonuniform methane distributions were required for the study. We chose this methane concentration based on various in situ methane concentration data collected in previous analytical works mostly by the sample bottling method and gas chromatography [9,11,13,20,21]. Although some amount of the methane in the XLPE may be released into the semiconductor before degassing starts, we assumed that the quantity was relatively low, and the amount of methane within the semiconductor layers did not meaningfully influence the general methane transport pattern or its degassing efficiency.

Based on the degassing chamber conditions described above, we applied an open boundary condition with zero methane concentration on the external surface of the cable. Due to the applied boundary conditions, the methane was released along the cable surface, and the flow direction on the cable surface was always normal to the cable surface, as shown in Equation (6).

(6) $$\left\{\begin{array}{cc} -n\cdot D\nabla c=0, & \mathrm{if}\;n\cdot u\geq 0\\ c=c_{0}, & \mathrm{if}\;n\cdot u<0 \end{array}\right.$$

where n is the unit vector defining the normal direction of the cable surface, u is the fluid diffusion velocity vector, and $c_{0}$ is the concentration of the fluid at the initial or the previous time step. In addition, the assumption regarding zero-methane concentration along the surface of the cables could be the best-case-scenario for in situ degassing practice, mostly due to the closed spaces located between the cable bindings on the drums. If the spaces are completely closed, the methane concentration may temporarily increase within the localized spaces, and this greater methane concentration may reduce the degassing efficiency nearby due to the lower concentration gradient. However, higher concentrations within such closed spaces can be minimized by adding sufficient additional spaces between the cable bindings to maintain an air circulation channel that is well connected to the outside of the drum.

**Figure 2:**
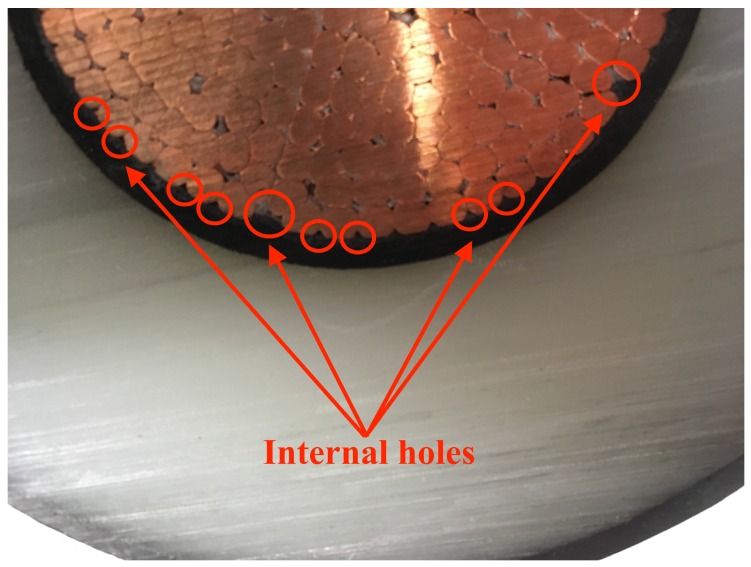
An XLPE-insulated power cable sample showing the internal holes along the interface of the ISC and the cable conductor and within the conductor.

Compared with the external boundary, however, previous studies assumed that the internal boundary along the contact area of the ISC and the conductor was in a no-flow boundary condition. This was due to the cable conductor that majorly consisted of a number of copper strands that had much lower fluid diffusion characteristics than the other polymer materials. Based on the significant difference of the diffusion properties between the conductor and surrounding polymer layers, applying a no-flow boundary condition along the interface seemed computationally reasonable and efficient, rather than including the whole conductor in the discretized finite element system. However, note that the no-flow boundary condition on the conductor surface can significantly exaggerate the cable condition in terms of methane diffusion in some cases. Although the geometrical characteristic was very difficult to capture and quantify, the cable conductor generally included some amount of additional free spaces, not only inside the conductor, but also along the contact area of the ISC and cable conductor, as presented in Figure 2. Furthermore, the space spreading along the interface between the ISC and the conductor may store some methane released from the polymer layers and also provide an important pathway for methane to flow into the inside of the conductor. In such cases, we determined that an internal hole boundary, i.e., a spatial combination of open and no-flow boundary conditions to allow a limited amount of methane flow into the conductor, may partly account for the effect of the flow channels on the degassing efficiency, and the details are described in the following section. Lastly, only the upper half of the cable was analyzed with no-flow boundary conditions assigned along the bottom boundary due to the symmetric geometry and boundaries of the cable representation shown in Figure 1b.

## 3. Case Studies and Results

Based on our observation of the cable degassing process, we noted that several geometric characteristics of the XLPE insulated power cables and their degassing conditions may significantly influence methane transport and degassing efficiency. Thus, such conditions had to be carefully selected and applied to the computational model to reliably examine their quantitative effect on the overall degassing efficiency. In this regard, possible controlling factors chosen for this research were: (1) the temperature-dependent diffusion coefficient of the polymer layers such as XLPE and semiconductor, (2) the free spaces distributed within the cable core and along the contact area between the inner semiconductor and cable core, and (3) the nonuniform distribution of the initial methane concentrations. To solve the governing equations described above, we applied a finite element method, and the entire cable geometry was discretized by applying the triangular mesh. The size of the mesh was generally controlled based on the thickness of the polymer layers and the existence of the internal holes along the conductor surface. For time discretization, an adaptive time stepping was applied so that the time step, Δt, was very small initially, but became greater as the degassing time increased. Using the finite element-based numerical model, we conducted a series of numerical studies on the degassing conditions, and the results are demonstrated in the following.

### 3.1. Temperature-Dependent Diffusion

We analyzed the effect of heat transfer within the cable and its influence on methane diffusion. At the beginning of the degassing process, the cable experienced a rapid temperature increase, up to 343.15 K (70 °C). This is the preferred degassing temperature generally accepted in the cable manufacturing industry [9,22,23], although this temperature can be increased further up to 353.14 K (80 °C) in some cases [9,13]. Such temperature changes may significantly influence methane diffusion because the diffusion coefficient of methane in XLPE ($D_{\mathrm{xlpe}}$) is heavily temperature dependent, as described in Equation (3).

**Figure 3:**
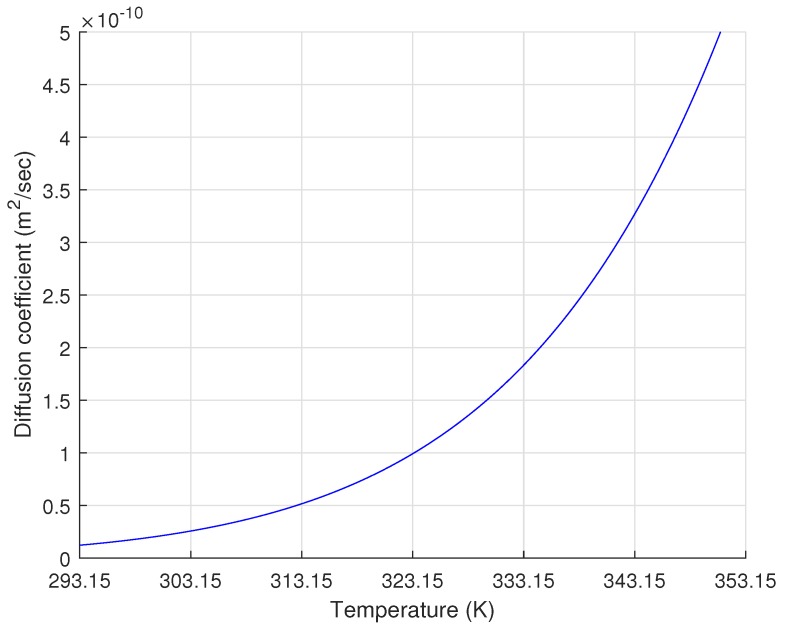
Diffusion coefficient of methane in the XLPE changed due to the temperature change from room temperature, 293.15 K (20 °C), to the maximum degassing chamber temperature, 353.15 K (80 °C).

In order to quantify specifically the relationship between temperature and diffusion in XLPE, Sun and Person [21] applied an analytical slab model and compared the simulation results with experimental data. Based on their results, the $D_{0}$ and $E_{a}$ of the XLPE were found to be 2.78 × 10${}^{2}$ m${}^{2}$/h and 55 kJ/mol, respectively. In this study, we applied these diffusion parameters to Equation (3) to estimate the temperature-dependent $D_{\mathrm{xlpe}}$ and the variation of $D_{\mathrm{xlpe}}$ due to the temperature change between 293.15 and 353.15 K, as shown in Figure 3. In Figure 3, the diffusion coefficient variation showed that a higher temperature caused a larger $D_{\mathrm{xlpe}}$, which eventually increased the degassing rate and vice versa. According to Andrews et al. [9], increasing the degassing temperature to 40 °C caused a reduction in the degassing time by an order of magnitude.

In contrast to the XLPE, the role of semiconductors in byproduct diffusion has received scant attention; there has been no quantitative analysis of the diffusion properties of semiconductors. This lack of attention is mostly due to the much smaller volume of semiconductors compared to other parts of an XLPE insulated power cable, such as the XLPE and cable conductor. Therefore, in this work, we assumed that the diffusion coefficient of the XLPE and semiconductors was the same over the applied temperature range, which may cause degassing efficiency variation compared to the degassing practice.

For the heat transfer analysis, we also included the cable conductor in the model owing to its highly deviating thermal properties next to the surrounding polymer layers. As summarized in Table 1, the major material of the cable conductor, copper, had greater density and thermal conductivity and less varying heat capacity compared to the cable insulation polymers. These highly deviating thermal properties of the cable conductor can greatly affect the heat transfer within the cable insulation layers, and therefore, this component must be taken into consideration in the model to robustly account for the effect of temperature change within the polymer layers.

**Figure 4:**
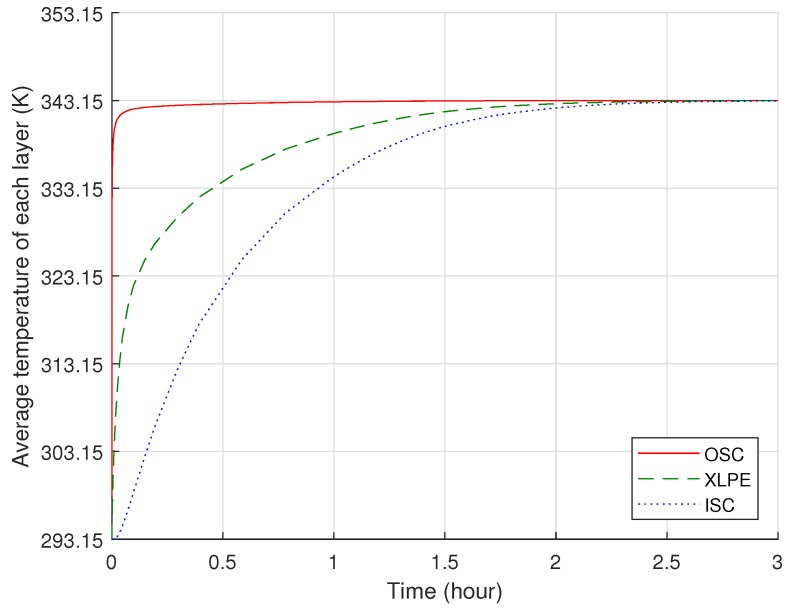
Average temperature variations of the OSC, XLPE, and ISC during the first three hours of degassing where the target degassing chamber temperature is 343.15 K.

For the initial condition of the heat transfer analysis, we first assumed that the cable temperature sufficiently decreased to atmospheric temperature, 293.15 K (20 °C), via the cooling process of the continuous vulcanization line [9,25,26]. Thus, in the computational model, we set the initial temperature of the entire cable at 293.15 K, and we applied a temperature of 343.15 K (i.e., the degassing chamber) only along the external boundary of the cable. Based on the thermal properties applied in the model, the boundary temperature was transferred through the XLPE and semiconductor layers over time, as shown in Figure 4. Due to the greater thickness of the XLPE compared to the semiconductor layers, a relatively longer time was required to increase the temperature of the entire XLPE compared to the semiconductors. The overall temperature of the cable, however, increased sharply up to the target temperature, and it only took less than three hours for all the polymer layers to reach the thermal equilibrium condition at 343.15 K. Specifically, average temperatures of the OSC, XLPE, and ISC sequentially reached the target degassing temperature after 1.48, 2.66, and 2.93 h of degassing, respectively.

**Figure 5:**
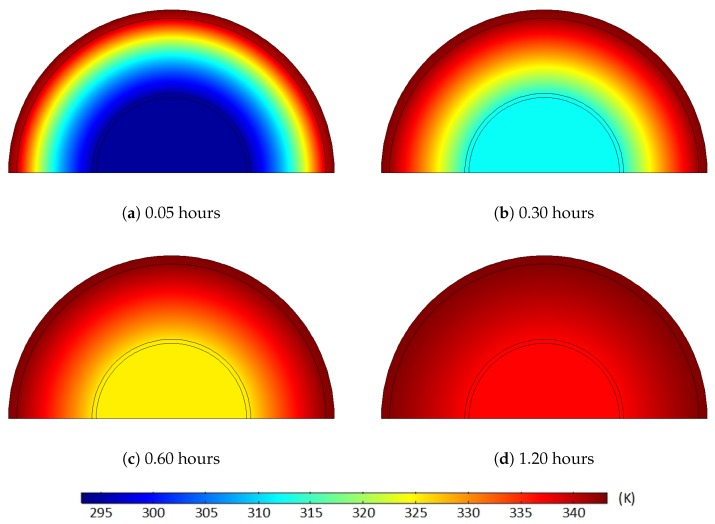
Temperature (K) of the cable at different degassing times where the target degassing chamber temperature is 343.15 K: (**a**) 0.05 hours, (**b**) 0.30 hours, (**c**) 0.60 hours, (**d**) 1.20 hours.

**Figure 6:**
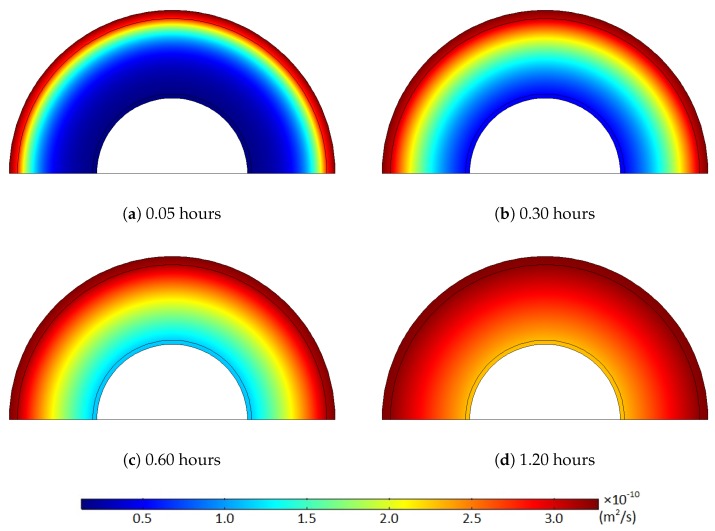
Diffusion coefficient (m${}^{2}$/s) of the XLPE and semiconductors, which corresponds to the temperature change where the target degassing chamber temperature is 343.15 K: (**a**) 0.05 hours, (**b**) 0.30 hours, (**c**) 0.60 hours, (**d**) 1.20 hours.

Using the temperature-diffusion correlation in Equation (3) and the diffusion properties from Sun and Person [21], we obtained the temperature variation and the corresponding diffusion coefficient distribution, as shown in Figure 5 and Figure 6. Figure 5 and Figure 6 clearly demonstrate that the diffusion coefficient rapidly rose due to the temperature increase. This diffusion coefficient distribution and its increase over time were directly applied in the computational diffusion model to account for the effect of heat transfer on the degassing process explicitly. However, the temperature-dependent diffusion coefficient was held constant in the latter part of the degassing process because the temperature equilibrium condition was acquired after the first three hours of degassing.

**Figure 7:**
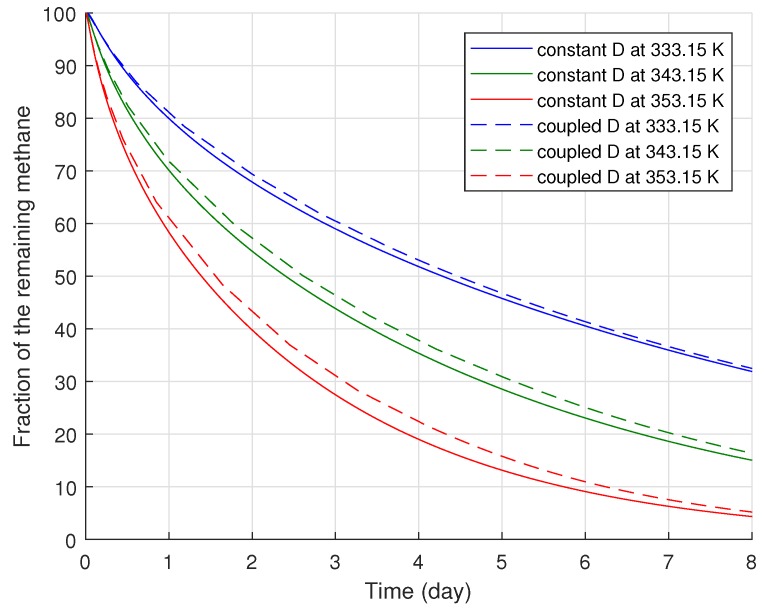
Degassing efficiency between constant and coupled diffusion cases with various degassing temperatures, 333.15, 343.15, and 353.15 K (equivalent to 60, 70, and 80 °C, respectively).

To compare the progress of methane degassing under various conditions, we estimated the proportional amount of the remaining methane over time compared to the total amount of methane stored in the fresh cable, i.e., the fraction of the remaining methane ($\int_{\Omega }c/\int_{\Omega }c_{t=0}$ in %). The methane amounts were numerically calculated by integrating the methane concentration over the total surface area of the polymer layers, Ω. In terms of the fraction of the remaining methane, we analyzed the methane degassing progress between two cases, namely the thermal-diffusion coupling case and the constant diffusion case; the results are presented in Figure 7. The thermal-diffusion coupling case indicated that the model accounted for the effect of the temperature-dependent diffusion coefficient, while the constant diffusion case was based on an assumption that the cable and degassing chamber temperatures were the same during degassing, and thus, the constant diffusion coefficient corresponding to the chamber temperature was only applied in the model regardless of the degassing time.

Figure 7 demonstrates that the temperature-dependent diffusion coefficient negatively affected the degassing efficiency, and the coupled diffusion coefficient varied with the degassing chamber temperatures. The greater the degassing temperature applied to the cable, the larger the deviation found in the degassing efficiency, because a longer time was required to heat the polymer layers up to a higher boundary temperature. Specifically, the maximum reductions of the degassing efficiency due to the coupled diffusion coefficient were approximately 1.73, 2.58, and 3.67% in terms of the fraction of the remaining methane where the degassing chamber temperatures were 333.15, 343.15, and 353.15 K, respectively. This result is quite reasonable because the constant diffusion case used only the maximum diffusion coefficient for the whole degassing time, while a relatively lower diffusion coefficient was applied to the thermal-diffusion coupled case at the beginning of the degassing process. Hence, such deviations in the degassing efficiency mainly arose during the initial degassing process when the cable temperature was lower than the degassing chamber temperature. Nevertheless, the overall difference in degassing efficiency between the constant and coupled diffusion cases was not significant. Although the maximum deviations were detected around the second day of degassing, the amounts decreased later as the degassing process continued. After eight days of degassing, for example, all the degassing efficiency deviations due to the coupled diffusion coefficient became less than 1.50%, regardless of the applied degassing chamber temperature.

In summary, using the temperature-dependent diffusion coefficient, our study showed how heat transfer in the cable affected the overall degassing process quantitatively. Based on these results, we saw that the effect of the temperature-dependent diffusion on the degassing efficiency was quite subtle, even with a wide variation of the degassing temperature that substantially affected methane diffusion. Due to the minor effect of temperature-dependent diffusion on methane degassing, we therefore did not include diffusion coefficient variations due to temperature change in the following subsections and considered only the constant diffusion coefficient at the target temperature at 343.15 K.

### 3.2. Free Spaces in the Conductor

**Figure 8:**
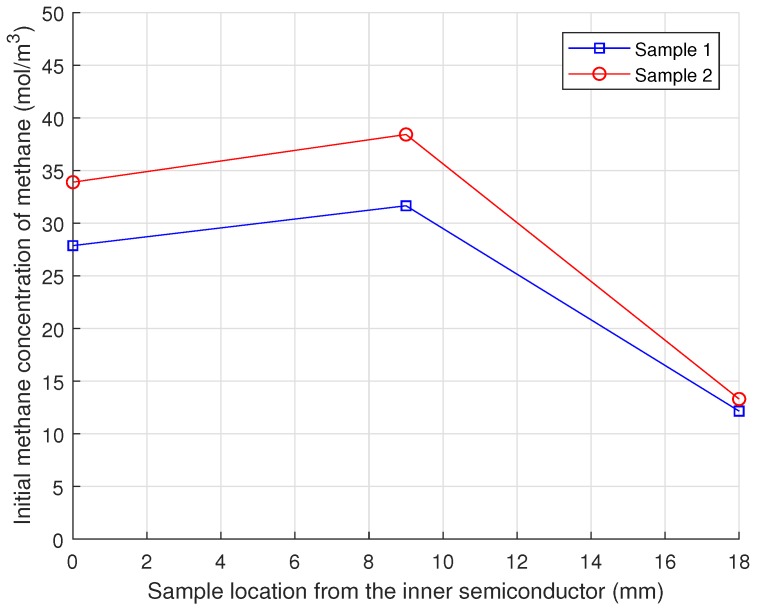
Nonuniform methane concentration datasets for a new XLPE insulated cable measured by Smedberg et al. [13].

Smedverg et al. [13] applied various experimental techniques to measure methane concentration distributions in a one-meter HV cable sample with an XLPE insulation of 15 mm in thickness. From the cable sample, the XLPE insulation was divided into three separate layers, near the ISC, in the middle of the insulation, and near the OSC, and several specimens were taken out from the three different layers to repeat the measurement. The observed concentration variation along the insulation thickness is presented in Figure 8. Although the concentration data in Figure 8 include some variability among the two sample sets, they clearly showed that the highest and lowest concentrations were found at the middle and near the OSC, respectively, and the concentration level near the ISC was intermediate between the maximum and minimum. The nonuniform distributions of methane in the XLPE strongly indicated that some amount of the methane was released during the cable manufacturing process or the sample preparation process.

This methane distribution pattern also delivered other key information on the byproduct diffusion in the cable system. Since the methane concentration near the ISC was lower than that in the middle of the XLPE, we noticed that some of the methane could be released into the air through the external boundary of the cable, as well as into the cable conductor via the internal boundary of the ISC. If the conductor completely blocked the methane flow, the highest methane concentration should be near the ISC. Although a small fraction of the methane in the XLPE could be transferred and stored in the ISC, the available free volume of the ISC (approximately 5% of the semiconductor bulk) was not large enough to admit the total amount of the methane released from the inner part of the XLPE (between the ISC and the middle of the XLPE). Based on this observation, we determined that the no-flow boundary condition did not account for the real diffusion condition of the conductor surface.

**Figure 9:**
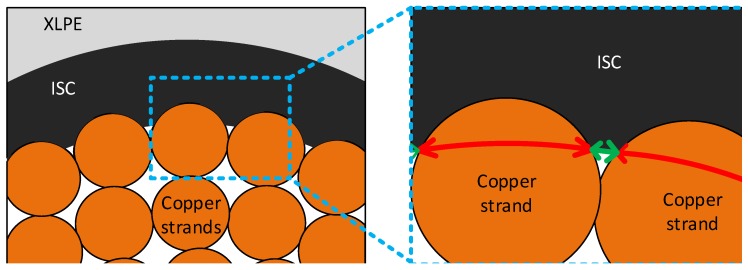
A schematic of an XLPE insulated power cable near the cable conductor, representing two boundary conditions, open (green arrow) and no-flow (red arrow) boundaries.

To allow some of the methane to be released into the cable conductor, we applied the internal hole boundary condition in the diffusion model as described in the previous section. The internal hole boundary condition was to consider the free spaces spreading along the contact area of the conductor and ISC. Therefore, the holes along the interface were computationally represented by a series of small open boundaries, while the rest of the contact area was completely blocked by the copper strands. For convenience of modeling and analysis, we also assumed that the sizes of all the open boundaries were the same, and the holes were uniformly distributed along the conductor surface. From our observation of the real EHV cable sample shown in Figure 2, a total of twenty-five open boundaries were assigned along the conductor surface. To quantify the size of the open boundaries, we additionally defined a proportional size relation of the open and no-flow boundaries as shown in Equation (7), and a schematic representation of the cable is demonstrated in Figure 9 (the green and red arrows in Figure 9 represent $l_{\mathrm{open}}$ and $l_{\mathrm{no}\text{-}\mathrm{flow}}$, respectively).

(7) $$l_{\mathrm{open}}=C_{l}l_{\mathrm{no}\text{-}\mathrm{flow}}$$

where $l_{\mathrm{open}}$ and $l_{\mathrm{no}\text{-}\mathrm{flow}}$ are the lengths of the open and no-flow boundaries, respectively, and $C_{l}$ is the unitless coefficient defining the length ratio between the open and no-flow boundaries. Thus, the size of the internal holes, $l_{\mathrm{open}}$, was controlled by changing $C_{l}$, and we assumed that $C_{l}$ varied from 1/10–1/90 in this simulation work. Regarding the suggested quantities in $C_{l}$, the boundary length data applied in the model is summarized in Table 2.

**Figure 10:**
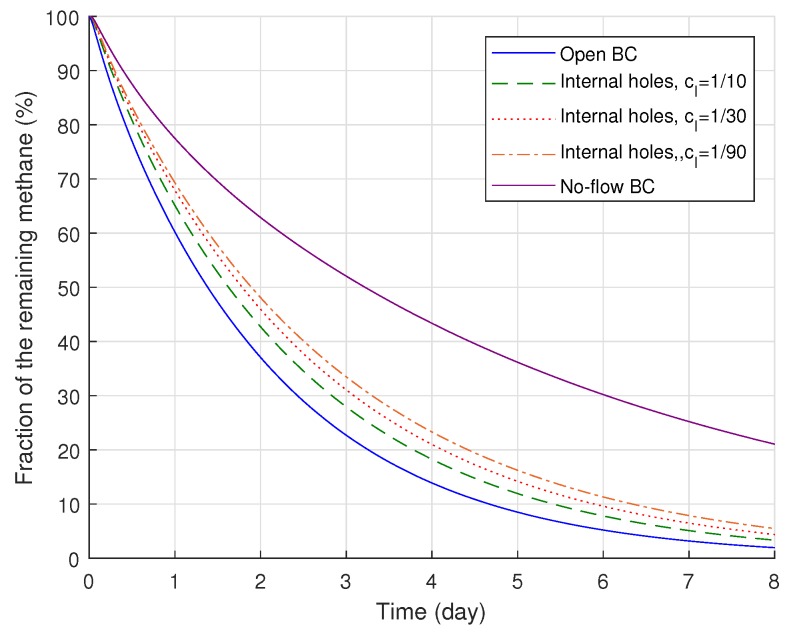
Degassing efficiency changes due to the boundary conditions for the cable conductor.

The effect of the interface holes on methane degassing and its comparison to two ideal cases (either open or no-flow boundary conditions applied along the whole conductor surface) are demonstrated in Figure 10. Figure 10 indicates that the greater the holes applied in the diffusion model by assigning greater $C_{l}$, the larger the degassing efficiency attained during the entire degassing time. However, the internal holes substantially increased the degassing efficiency even when the internal holes were very small, and all the degassing efficiency curves in Figure 10 are much closer to the open boundary case compared to the no-flow boundary case. Although we found the maximum deviation between the open boundary and internal hole boundary conditions after three days of degassing, the remaining amount of methane with the internal holes became even closer to the open boundary case (blue line in Figure 10) as degassing continued. After eight days of degassing, the remaining volumes of methane eventually became very close to that for the open boundary case, and the deviation between the cases ended up at 0.97, 1.75, and 2.48%, where $C_{l}$ = 1/10, 1/30, and 1/90, respectively.

**Figure 11:**
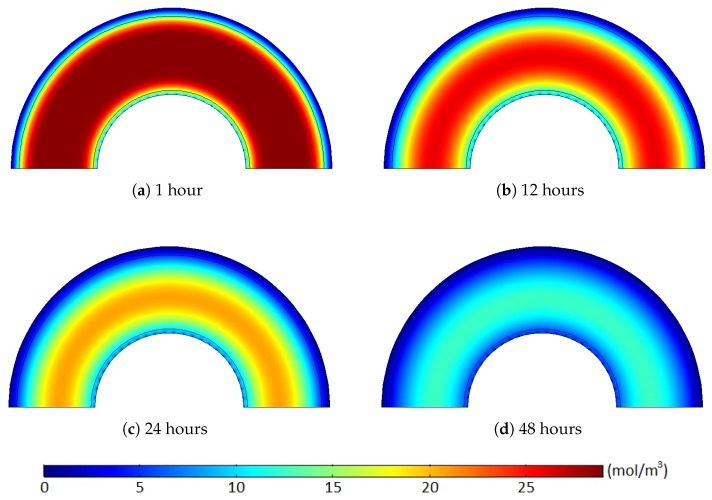
Methane concentration ${(\mathrm{mol}/m}^{3})$ over time with the internal holes where the target degassing chamber temperature is 343.15 K and $C_{l}=1/90$. (**a**) 1 hours, (**b**) 12 hours, (**c**) 24 hours, (**d**) 48 hours.

**Figure 12:**
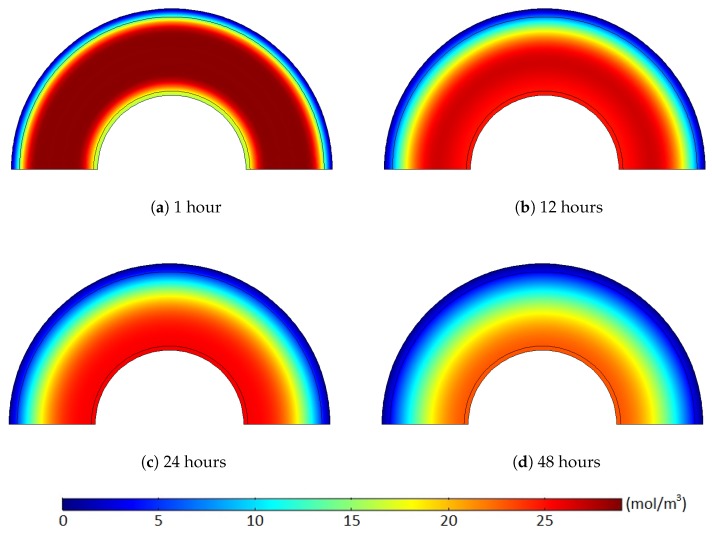
Methane concentration ${(\mathrm{mol}/m}^{3})$ over time with the no-flow boundary condition where the target degassing chamber temperature is 343.15 K and $C_{l}=1/90$. (**a**) 1 hours, (**b**) 12 hours, (**c**) 24 hours, (**d**) 48 hours.

Figure 11 and Figure 12 show the methane concentration change patterns within the polymer layers with either the internal hole or the no-flow boundary condition for the conductor surface.

For the case with the internal holes, the greatest methane concentration was located near the middle of the XLPE layer during the whole degassing process because the methane was simultaneously released through both the external boundary and the internal holes. For the case with the no-flow boundary condition, however, the methane can only be degassed through the external boundary. Thus, the location of the maximum methane concentration was gradually shifted from the middle of the XLPE to the inner boundary of the cable during a relatively short time (less than 24 h). Moreover, some methane in the inner part of the XLPE was also transferred to the ISC because of the zero-methane concentration initially applied within the ISC. This amount of methane does not affect the concentration gradient of the entire cable. It is very obvious that the greater degassing efficiency with the internal hole condition was mainly due to the additional methane release through the internal holes, but this reduced concentration gradient with the no-flow boundary condition can also be an additional source of the the degassing efficiency reduction.

In summary, we demonstrated the effect of internal holes on the methane degassing efficiency under various sizes of the internal holes. Our results showed that the presence of internal holes substantially increased the overall degassing efficiency even when the hole size was extremely small. Therefore, the degassing efficiency measured with the internal hole condition was generally much higher than for the case with no-flow boundary conditions for the conductor; this was observed for most previous cable degassing studies and was due to the substantially lower diffusion property of the pure copper. However, the simulation results showed that the no-flow boundary condition for the cable conductor overly simplified the role of cable conductor on the methane degassing from the cable. The internal holes distributed near the conductor surface substantially contributed to the methane degassing. Without accounting for the holes, accurate estimation of the methane degassing is barely possible, especially for the methane exchange between the cable conductor and the surrounding polymers during the degassing. Due to the importance of the internal holes as for the methane degassing phenomena, the next parametric study regarding the methane distribution pattern in the following subsection was also carried out with the internal hole boundary condition.

### 3.3. Nonuniform Methane Concentration

As shown in the previous subsection, the initial distribution of methane within the XLPE was not simply uniform. Such a nonuniform distribution of the initial methane concentration directly influenced the methane diffusion efficiency within the cable due to the different spatial distributions of the concentration gradient compared to that in the uniform distribution case. In this subsection, therefore, we apply the nonuniform methane concentration as the initial condition of the degassing analysis under various boundary conditions and compare its effect on the degassing efficiency against the case with a uniform methane concentration.

To compare the effect of methane distribution types on the degassing efficiency directly, the same degassing simulations as for the initial condition were conducted with either a uniform or nonuniform methane distribution, while the total amount of methane at the beginning of the degassing was the same regardless of the methane distribution types. From the nonuniform methane distribution data presented in Figure 8, the average values were firstly measured at each XLPE location, and the averaged bi-linear distribution was applied similarly to the initial methane concentration of the nonuniform distribution case. Later, the total methane concentration in the nonuniform distribution case was divided by the surface area of the XLPE to estimate the average methane concentration over the entire XLPE domain for the uniform distribution case. These two different methane distributions from the same total of methane were separately applied to the diffusion model, and fractions of the remaining methane were analyzed with regards to the additional boundary conditions on the conductor surface.

**Figure 13:**
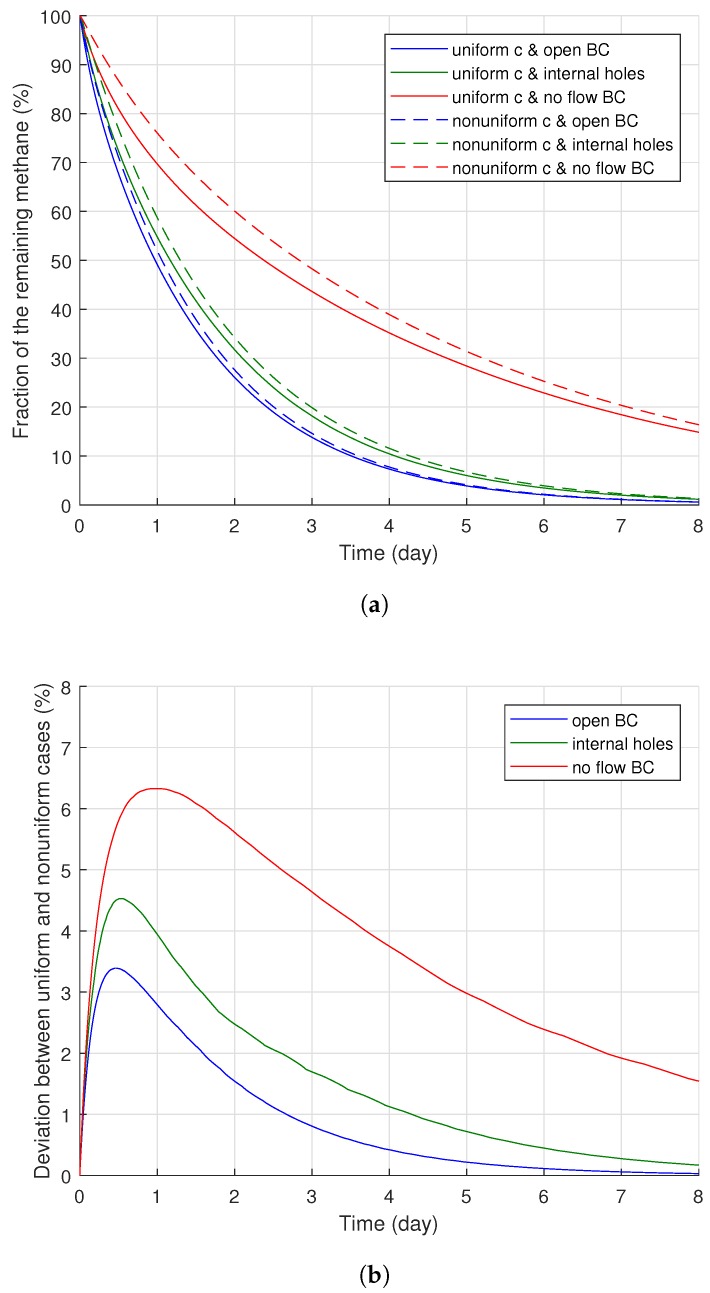
Methane degassing data with either a uniform or a nonuniform methane distribution. (**a**) Remaining methane changes over time; (**b**) deviation of the methane between uniform and nonuniform cases.

Figure 13a displays the methane degassing efficiency results for the different types of methane distribution. Generally, the nonuniform methane distribution case presented less degassing efficiency than the uniform methane distribution case, regardless of the boundary condition applied for the cable conductor. However, the extent of deviation of the degassing efficiency between the two cases was influenced by the degassing time and the boundary conditions, as shown in Figure 13b. The deviations initially increased from the beginning of the degassing process for a relatively short period (less than one day), but later, they gradually decreased as the degassing continued. Specifically, the maximum quantities of the deviation were 6.30, 4.53, and 3.39% after 0.97, 0.52, and 0.44 days of the degassing with no-flow, and they began to decrease and eventually became significantly minor quantities (1.54, 0.17, and 0.03%, respectively) after eight days of degassing.

**Figure 14:**
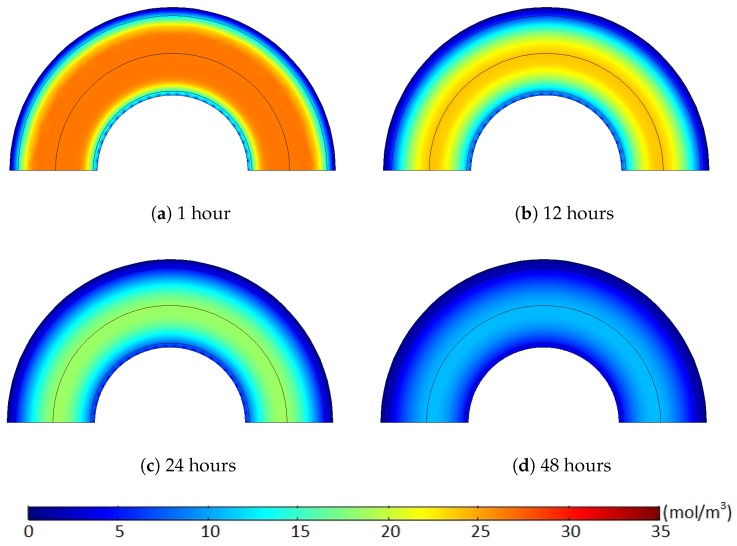
Methane concentration (mol/m${}^{3}$) over time where the initial methane concentration is uniform. The degassing temperature is 343.15 K and $C_{l}=1/90$. (**a**) 1 hours, (**b**) 12 hours, (**c**) 24 hours, (**d**) 48 hours.

**Figure 15:**
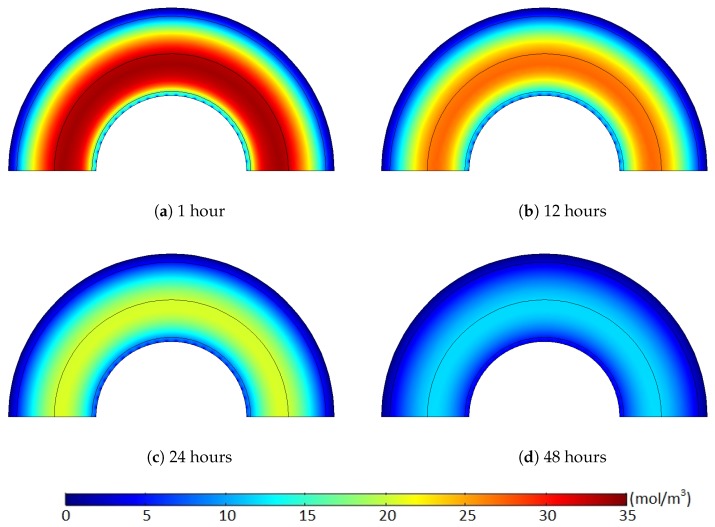
Methane concentration (mol/m${}^{3}$) over time where the initial methane concentration is nonuniform. The degassing temperature is 343.15 K and $C_{l}=1/90$. (**a**) 1 hours, (**b**) 12 hours, (**c**) 24 hours, (**d**) 48 hours.

Such a time-dependent deviation quantity change was based on the methane distribution and corresponding concentration gradient along the polymer layers. An initial amount of the degassed methane was mostly from narrow areas near the external boundaries due to the relatively high concentration gradient (relatively short distance and high concentration change), and the nonuniform methane distribution case initially included less methane within the region compared to the uniform distribution case, as shown in Figure 14 and Figure 15. Hence, the degassing efficiency of the nonuniform methane distribution case was lower than that of the uniform methane distribution case, and the deviation between the cases increased during the initial degassing stage.

After some point, however, this methane transport pattern gradually diminished once most of the methane within the limited regions was released from the cable. While the initial degassing process continued, for the uniform methane distribution case, the methane concentration level in the middle of the XLPE was mostly maintained constant due to the extremely minor concentration gradient within the middle of the XLPE layer (see the layer in orange shown in Figure 14). Therefore, methane transport was barely possible within the uniform concentration region until the front end of the methane concentration reduction approached near the area to initiate some concentration gradient. Thus, this passive diffusion characteristic of the methane in the middle layer did not support the degassing efficiency for the initial stages of the degassing process. In contrast to the uniform case, the nonuniform methane distribution maintained a relatively constant level of the concentration gradient distributed over the whole XLPE layer. As shown in Figure 15, therefore, all the methane within the XLPE actively transferred during the entire degassing time. This continuous methane diffusion within the whole XLPE increased the degassing efficiency and, thus, reduced the degassing efficiency deviation later in the degassing process as presented in Figure 13b and Figure 14. In addition to the general comparison of the methane distribution, the deviation between the uniform and nonuniform distribution patterns strongly depended on the internal boundary condition for the conductor as well. As presented in Figure 14b, the largest and the smallest deviations were found where no-flow and open boundary conditions were applied for the conductor surface, while the internal hole boundary conditions led to the intermediate deviation, albeit closer to the case with the open boundary condition. Based on the deviation data obtained under different boundary conditions and the methane transport characteristics, the more difficult the methane diffusion condition applied to the cable degassing analysis, the larger the deviation between the uniform and nonuniform distribution cases.

In conclusion, the nonuniform methane distribution case obviously showed less methane degassing efficiency during a relatively short time of the degassing, and the internal boundary condition for the conductor also significantly influenced the degassing efficiency. In the long term (7–8 days), however, the difference of the remaining methane concentrations due to the two methane distribution types greatly decreased over time. Based on these observation, we can thus confirm that the nonuniform methane distribution for a fresh cable was an important modeling parameter for cable degassing evaluation and analysis. The degassing efficiency calculation with a uniform methane distribution may result in substantial error on degassing efficiency, especially during the initial stages of degassing. However, the amount of error due to the uniform methane distribution became smaller with the degassing time.

## 4. Conclusions

We performed a series of numerical studies to discover the key modeling parameters and boundary conditions related to degassing of the byproduct (CH${}_{4}$) from the XLPE insulated HV power cables. We used the two-dimensional diffusion model based on Fick’s law under various degassing conditions, which we selected based on the methane transport pattern and degassing efficiency. We successfully demonstrated how different degassing conditions, i.e., (1) the temperature-dependent diffusion coefficient, (2) the existence of internal holes along the external surface of the cable conductor, and (3) the nonuniform initial concentration of the methane, can affect methane transport behavior and degassing efficiency. We precisely applied these degassing conditions to the model with technically reliable assumptions, and below, we summarize our findings based on these results.

- The temperature-dependent diffusion coefficient did not greatly affect overall methane release from the cable because the cable temperature increased very rapidly for the first couple of hours of the degassing process.
- The change of the boundary condition for the cable conductor significantly influenced the degassing process. Among the various boundary conditions, the internal hole condition played a critical role in the byproduct degassing, even if the hole sizes were extremely small. Compared to the no-flow boundary condition, the internal hole case showed a much higher degassing efficiency, which required less time to release the proper amount of methane from the cable. We hypothesize that the holes provided a free space to store the methane and also a flow pathway for the methane transferred into the conductor.
- Nonuniform distribution of the initial methane concentration was also a key feature that must be accounted for in the degassing analysis. Generally, the nonuniform distribution case had less degassing efficiency than the uniform distribution case, while the effect on the degassing may vary greatly, depending on the degassing time and the internal boundary conditions for the cable conductor.

This research demonstrated how the computational model was used to analyze the complex byproduct transport process within the polymer insulation of cables. The presented degassing data under various conditions may contribute the cable design and the degassing chamber condition for enhancing the degassing efficiency.

## Figures and Tables

**Table 1:** Properties of cable materials for heat transfer analysis [22,23,24].

Property	Copper	XLPE	Semiconductor
Density, ρ (kg/m${}^{3}$)	8960	920	1150
Thermal conductivity,			
*k* (W/m K)	401	0.22–0.28	0.57–0.65
Specific heat capacity,			
$C_{p}$ (J/kg K)	385	135–983	115–433

**Table 2:** Lengths of open and no-flow boundaries with respect to $C_{l}$.

$C_{l}$	$l_{\mathrm{open}}$	$l_{\mathrm{no}\text{-}\mathrm{flow}}$
1/10	0.207 mm	2.056 mm
1/30	0.073 mm	2.189 mm
1/90	0.025 mm	2.237 mm

## Abbreviations

The following abbreviations and notations are used in this manuscript:

AP and CA	Acetophenone and cumyl alcohol
BC	Boundary condition
*c*	Concentration of fluid under consideration
$c_{t=0}$	Concentration of fluid under consideration in fresh cable
$C_{l}$	Unitless coefficient defining the length ratio between the open and no-flow boundaries
$C_{p}$	Specific heat capacity
*D*	Diffusion coefficient
DCP	Dicumyl peroxide
DSC	Differential scanning calorimetry
$D_{\mathrm{xlpe}}$	Diffusion coefficient of methane in XLPE insulation
$D_{0}$	Dimensionless prefactor defined in the Arrhenius Equation
$E_{a}$	Activation energy
FTIR	Fourier transform infrared spectroscopy
GC	Gas chromatography
HPLC	High-pressure liquid chromatography
HV and EHV	High-voltage and extra-high-voltage
ISC and OSC	Inner and outer semiconductors
*J*	Diffusion flux
*k*	Thermal conductivity
$l_{\mathrm{open}}$ and $l_{\mathrm{no}\text{-}\mathrm{flow}}$	Lengths of the open and no-flow boundaries
PE	Polyethylene
q	Heat flux
*R*	Universal gas constant
*t*	Time
*T*	Temperature
TGA	Thermo-gravimetric analysis
XLPE	Crosslinked polyethylene
ρ	Density
Ω	Surface area of the polymer layers
